# Gender-based violence in Pakistan and public health measures: a call to action

**DOI:** 10.2471/BLT.21.287188

**Published:** 2022-06-06

**Authors:** Azza Sarfraz, Zouina Sarfraz, Muzna Sarfraz, Zul Qarnain

**Affiliations:** a Department of Pediatrics and Child Health, Aga Khan University, Stadium Road, Karachi 74800, Pakistan.; b Department of Research and Publications, Fatima Jinnah Medical University, Lahore, Pakistan.; c Medical College, King Edward Medical University, Lahore, Pakistan.; d Department of Biological and Biomedical Sciences, Aga Khan University, Karachi, Pakistan.

Gender-based violence refers to harmful acts aimed at individuals based on gender, specifically against women – one of the most pervasive human rights violations globally.^1^ A direct association exists between gender-based violence and the health status of women, such as physical injuries, permanent disabilities, psychological disorders, suicide, sexually transmitted diseases, unwanted pregnancies, female feticide, unsafe abortions and death.^2^ Pakistan has a population of over 229 million, and ranked as the fourth most dangerous country for women in 2021.^3^ Thirty-two per cent of women (48/150) randomly selected from health facilities in Karachi, Pakistan have experienced physical violence, and between 70% and 90% of married women have experienced abuse from their spouses at any time in their lives according to a survey on 1000 women in Punjab, Pakistan.^4^ Violence by spouses and other male relatives against women is the most widespread form of violence in Pakistan.^4^ In Punjab, the most populous province in Pakistan, the conviction rate in rape cases was 4% (99/2343) in 2016,^5^ and government resources to support the victims are scarce.^4^ Many recent incidents of gender-based violence resulting in major physical injuries and even death have mobilized women in Pakistan;^6^ however, the collective trauma of women in Pakistan might cause a reluctance to seek help due to the lack of appropriate services and community support.^2^ Here, we call to action to mitigate gender-based violence, the consequences of which should be recognized as a humanitarian emergency in Pakistan – a country where women are not safe. 

**Fig. 1:**
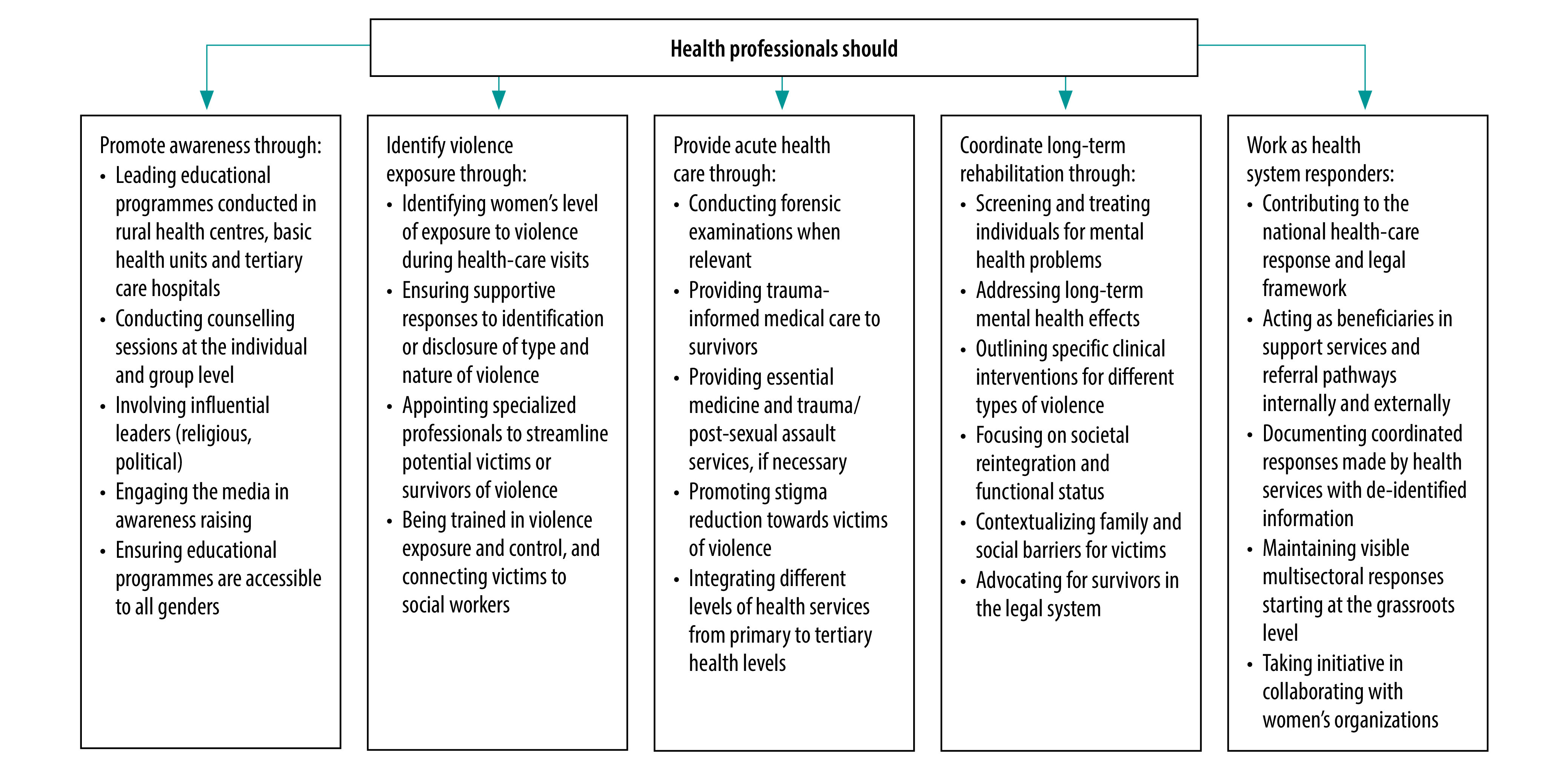
Proposed health-care response to violence against women in Pakistan

We adapted a framework from the *National policy on ending violence against women and girls*,^7^ considering the three-tiered model of public health infrastructure in Pakistan (Fig. 1). 

Promoting awareness of different forms of violence against women across key stakeholders is necessary, including different tiers of health-care service, media outlets, religious leaders and the public. Existing research and media sources report women’s status in Pakistan; however, these reports have been restricted to urban women. Yet in Pakistan, two thirds of all women live in rural areas where human resources, technology and funding are typically restricted, highlighting a major gap in intervention measures. Ingrained practices towards women within the Pakistani culture are often those of oppression, possession, objectification and use as bargaining tools (for example, made to marry in families with ongoing disputes as a peace offering and conflict resolution mechanism). These practices take place in rural settings more often because rural women usually have a lower social, economic and political status. Cities such as Karachi, Lahore and Islamabad, overall, have less discriminatory gender systems due to higher class influences and greater education and employment autonomy for women. Such is not the case for rural women, despite their contribution to farming and households, as these tasks are not formally paid. Consequently, activities such as awareness campaigns are more likely to be effective in urban areas. Awareness at a grassroots level in rural areas may not be entirely feasible due to barriers to access and cultural restrictions, and would require a fundamental sociopolitical change at the domestic, societal and provincial levels through non-discriminatory education and employment opportunities. Gender-based violence against women is rooted in the patriarchal interpretations of religion in Pakistan; religious principles on pre-existing behavioural codes of Pakistan contribute to the alarmingly high rates of gender-based violence. However, we believe such violence is a cultural issue.

Preventing violence across all six provinces and territories of Pakistan is necessary. The gaps in gender-based violence prevention are lack of education, scarce safe employment opportunities, societal pressures, inadequate public health-care resources to address gender-based violence, suppressed freedom of expression and minimal legal action against women’s rights violations due to cultural practices. In Pakistan, domestic violence is not considered a matter for legal courts because it is viewed as a private issue, and therefore is not prioritized in the assessment of policies, medico-legal interventions or policy changes. Recently, a legal bill against domestic violence, which was received with huge criticism, was put forward to the National Assembly by the human rights ministry. The bill highlighted women’s rights to safety and demanded that perpetrators be punished by law; however, the bill did not pass. 

First-level health-care facilities in Pakistan, that is, basic health units and rural health centres are mostly staffed by low-skilled male workers, who may have deep-rooted social biases. Encounters with the health system can discourage victims of gender-based violence from seeking help and makes screening victims or individuals at risk challenging. Support is needed from human rights organizations and Pakistan’s federal and provincial governments to adopt and implement measures against gender-based violence. Although Pakistan’s national action plan on human rights includes the protection of women as a vital component of its agenda, the plan is not implemented, particularly in pockets characterized by patriarchal systems and religious extremism.^3^ Cultural challenges also prevent women from reaching out for help. The United Nations reports that as of April 2021, only 1.5% (5870/391 364) of police officers are female in Pakistan.^8^ All law enforcement agencies should be monitored closely for gender-discriminatory behaviour, and the incorporation of a higher proportion of females in these institutions should be encouraged.

Taking action against perpetrators of gender-based violence is crucial. A national health-care response incorporating a legal framework with clear referral pathways, for instance within health services, the police force or financial institutions, must be developed and implemented. Such a framework should consider intracountry cultural barriers that vary by province and/or territory. Regular surveys in rural villages will help provincial and federal governments gain insight into the severity of gender-based violence. As a first-line response, an adequately trained team must include health-care workers, and police officers must have access to these basic health units and rural health centres to monitor and act against gender-based violence. However, a risk of harm to female health workers who deal with gender-based violence in these units and centres exists, because of the overall patriarchal system. The government needs to acknowledge the risk against female health workers and address it through the criminal justice system. Although a guideline booklet was published in 2019 by the Pakistan Health Knowledge Hub and the Ministry of National Health Services Regulations and Coordination, the medico-legal framework needs an implementation plan and to be used outside urban and peri-urban settlements.^9^ Documentation of the response to violence against women (medical treatment received, legal services given) is crucial for reducing gender-based violence prevalence as perpetrators may fear punitive action. Collaborative efforts from shelter groups and nongovernmental organizations for women operating in remote and rural settings as first-line correspondents are also vital.

Acute care for gender-based violence victims must be considered. An integrated health-care approach must encompass medical care, legal services, psychological counselling and rehabilitation, as well as access to shelter homes. Most gender-based violence victims may end up in slums and other informal settlements without access to shelter homes.^10^ Resources are scarce at the basic health units and rural health centres level, requiring special federal budget allocation to expand health-care infrastructure and shelter homes for gender-based violence victims. Public health measures should include free forensic examinations whenever relevant, and help victims collect evidence to strengthen their fight against gender-based violence cases within the criminal justice system. Unfortunately, most basic health units, rural health centres, nongovernmental organizations and shelter homes do not have, or have insufficient, forensics officers. DNA (deoxyribonucleic acid) testing, the main evidence that the criminal justice system demands from plaintiffs in Pakistan, is only available in a few large tertiary care centres in urban settlements. In addition, introducing trauma-informed care in the public health-care system is needed, to gain basic understanding of how trauma impacts the lives of women seeking health care.^11^ Universal trauma precautions and trauma-specific care can be provided to support the establishment of trust between victims and medico-legal personnel. Primary health-care service guidelines may consider the trauma-informed care pyramid to ensure patient-centred care.^12^ Finally, victims who experience ostracization should be reintegrated in social networks, for example by funding community-based interventions through nongovernmental organizations. Vulnerable groups such as unemployed women or married female children require concerted efforts for countering ostracization.

Similarly, providing long-term rehabilitation to survivors of gender-based violence is necessary. The focus of long-term rehabilitation measures should be the health of women, promoting fully functional status and social reintegration by countering mental health sequelae and stigma. Screening and treating gender-based violence survivors for long-term mental and physical health outcomes, as well as rehabilitation through multisectoral approaches, including education, employment opportunities and legal support, are required. However, most mental illnesses caused by gender-based violence are unreported, especially in less developed provinces, due to social stigmas such as fear of divorce and rejection among women. Acid-attack victims are attacked because they are women, and perpetrators are not held accountable. Rural health-care facilities do not have reconstructive surgery units; introducing rehabilitation for all victims of physical disfigurement is therefore also needed. 

Pakistani authorities face critical challenges because public health organizations work in silos. The under-representation of women in the political, educational and legislative spheres normalizes gender under-representation in decision-making. The medico-legal system should implement a zero-tolerance policy towards gender-based violence. However, these actions will remain incomplete if the cultural norms that have normalized gender-based violence are not challenged across the country. Incidents of gender-based violence are seldom a one-time occurrence and are a recurring and endemic phenomenon. In Pakistan, the impact and consequences of gender-based violence are not entirely understood because they are either concealed or not discussed. We call to end violence against women, in Pakistan and globally, but doing so requires the adoption and implementation of stronger and consistent laws, policies and strategies focused on preventing and responding to violence against women.
